# Differences in Sequential Eye Movement Behavior between Taiwanese and American Viewers

**DOI:** 10.3389/fpsyg.2016.00697

**Published:** 2016-05-20

**Authors:** Yen-Ju Lee, Harold H. Greene, Chia W. Tsai, Yu J. Chou

**Affiliations:** ^1^Department of Psychology, University of Detroit Mercy, DetroitMI, USA; ^2^National Dong Hwa UniversityHualien, Taiwan

**Keywords:** facial emotion, culture, attention, eye movements

## Abstract

Knowledge of how information is sought in the visual world is useful for predicting and simulating human behavior. Taiwanese participants and American participants were instructed to judge the facial expression of a focal face that was flanked horizontally by other faces while their eye movements were monitored. The Taiwanese participants distributed their eye fixations more widely than American participants, started to look away from the focal face earlier than American participants, and spent a higher percentage of time looking at the flanking faces. Eye movement transition matrices also provided evidence that Taiwanese participants continually, and systematically shifted gaze between focal and flanking faces. Eye movement patterns were less systematic and less prevalent in American participants. This suggests that both cultures utilized different attention allocation strategies. The results highlight the importance of determining sequential eye movement statistics in cross-cultural research on the utilization of visual context.

## Introduction

Western Psychology theories have traditionally assumed universality in human cognitive processes (e.g., Shepard, 2004). Cross-cultural research has provided exceptions to universality, making it unrealistic to assert that all human groups utilize similar cognitive processing strategies (see Nisbett et al., 2001; Nisbett, 2003; Nisbett and Miyamoto, 2005). Perceptual research evidence suggests that people who live in Western countries usually process the visual world in analytical ways that emphasize salient objects, and people living in East Asian countries usually process the world in holistic ways that incorporate context objects with salient objects (see Nisbett et al., 2001; Nisbett and Miyamoto, 2005, for a review). If human groups differ in the way they fundamentally process the world, determining the underlying cognitive mechanisms is important for understanding and predicting diverse/international responses to identical situations. Cognitive mechanisms that may be influenced by culture include those involved in attention, information encoding and retrieval, and selective reporting of information about the world. In the present study, the mechanism of interest was attention.

### Cross-Cultural Differences in Attention to Context

Cultural differences have been found to moderate activation in brain regions involved in the control of attention (Hedden et al., 2008). Hedden et al. (2008) instructed participants to judge the length of a line inside a frame, while ignoring or attending to the frame. Brain imaging results (fMRI) showed that European American students had high activation levels in attention control areas when instructed to attend to the line and its frame (i.e., when instructed to be holistic in their attention to the visual stimulus). In contrast, East Asian students who had recently arrived in the USA, had high activation levels in these same brain areas when instructed to ignore the frame (and attend solely to the line). The findings indicate that there was a greater demand for attentional control when East Asian participants utilized a (non-preferred) independent/analytical cognitive processing style, and when European American participants utilized a (non-preferred) holistic cognitive processing style (Hedden et al., 2008).

Differences in attentional control between East Asians and Westerners are also apparent in eye movement behavior (Chua et al., 2005; Masuda et al., 2008, 2012). In Chua et al.’s (2005) study, Westerners (i.e., European American graduate students) and East Asians (i.e., international Chinese graduate students) were presented scenes that contained a focal object within a background context (e.g., a centrally placed fighter jet flying above mountainous terrain on a cloudy day). Each scene was presented for 3 s only, thus optimizing participants’ use of eye movements to seek information (rather than to redundantly scan the scenes). While both groups of participants fixated the background context more than they did the focal object in presented scenes, East Asians made more fixations than Westerners on the background context. For Westerners, fixations were longer on the focal object than on the background context. In contrast, for East Asians average fixation durations on focal object and background context were comparable, suggesting comparable time in acquiring information with each fixation from both parts of the presented scenes.

Whereas Chua et al.’s (2005) findings support the theory that East Asians are more holistic than Westerners in their overt allocation of attention (i.e., with eye movements) to visual scenes, some scene perception studies (e.g., Rayner et al., 2007; Evans et al., 2009) have failed to replicate the findings. East Asians in the scene viewing studies (Chua et al., 2005; Rayner et al., 2007; Evans et al., 2009) were international students in USA. Miyamoto et al. (2006) have reported that American students in USA and Japanese students in Japan, had a greater sensitivity to background scene information after both sets of students were pre-exposed to Japanese scenes. Evidently, even short-term visual experiences may influence subsequent attention performance in a scene-viewing task. Thus, it is reasonable to expect East Asians studying in USA (albeit for a short while) to exhibit western-like overt attention to scene context. For studies with contradictory findings (Chua et al., 2005; Rayner et al., 2007; Evans et al., 2009), it has been suggested that instructions requiring relatively passive responses may have promoted the execution of eye movements that were strongly influenced by local scene contrasts (Senzaki et al., 2014). The dominance of local contrast may have led unpredictably to culture-specific, or culture-invariant eye-movement behavior, depending on study-specific stimulus configurations and participants’ interpretation of instructions. Interestingly, Senzaki et al. (2014) demonstrated that when the instruction was to describe animated scenes, as opposed to passively rating them for likeness (Chua et al., 2005; Evans et al., 2009), or passively waiting for a scene-recognition test (Rayner et al., 2007), cultural differences in eye movements were evident between Japanese and Canadian participants. No cultural difference was evident for passive viewing for the same scenes. Hence, cultural differences in eye movement behavior may be more evident when participants are required to engage in active top-down processing of viewed scenes (Senzaki et al., 2014).

Unlike scene-viewing studies, facial emotion studies with contextual content (Masuda et al., 2008, 2012) have been consistent in finding cultural differences in eye movement behavior. One explanation for this may be fundamental differences in scene and face processing. For example, brain imaging findings have revealed that, whereas scene processing is mediated by the para-hippocampal place area (PPA; Epstein and Kanwisher, 1998), face processing is mediated by the fusiform face area (FFA; Tong et al., 1998; Grill-Spector et al., 2004). Another explanation is that the decoding of facial emotions necessarily involves more active top-down processing than local-contrast processing (see Senzaki et al., 2014, for the role of top-down processing in effecting cultural differences in eye movement behavior). Finally, unlike open-ended scene-viewing tasks where participants are free to prioritize attention to detail, in facial emotion studies, the goal of all participants is controlled: Irrespective of previous visual experiences which may influence attention allocation (e.g., Miyamoto et al., 2006), everyone’s primary task is to judge actively the emotion on the same focal face. Thus, facial emotion tasks provide a useful tool for studying cultural differences in attention to context.

The ability to correctly interpret facial emotions in social contexts is important in multicultural interactions (Masuda et al., 2008, 2012). In Masuda et al.’s (2008) study, East Asians (Japanese students in Japan) and Westerners (Anglophones living in Japan at the time of the study) viewed and judged the emotion on the focal cartoon character that was flanked on either side by emotion-bearing cartoon characters. Viewing times were left to the discretion of the participants, but only the first 3 s of viewing were analyzed (comparable to Chua et al., 2005 3-s stimulus presentations). East Asians tended to spend less time than Westerners viewing the focal face, and more time than Westerners viewing the flanking faces. Consistent with Masuda et al.’s (2008) findings, Masuda et al. (2012) found that East Asians (i.e., Asian international students and Japanese students in Japan) allotted more fixations to flanking faces than European and Asian Canadians (i.e., Westerners). East Asians also spent longer times viewing flanking faces than Westerners. Together, eye movement studies (Chua et al., 2005; Masuda et al., 2008, 2012) suggest with some caution (e.g., Rayner et al., 2007; Evans et al., 2009) that East Asians are more holistic than Westerners in the way they overtly attend to visual information. Time course analyses in the scene-viewing, and facial emotion studies have suggested that the holistic attention to detail by East Asians may start as early as 420–1000 ms of the onset of a visual scene (Chua et al., 2005; Masuda et al., 2008).

### Unresolved Issue

Although previous studies on cultural differences in attention to context have addressed *where participants looked* (e.g., focal object vs. context objects), *when* on average, they initiated a shift of interest from the focal to the context objects, and *how long* they dwelt on focal vs. context objects, they have not addressed *how* participants *continually* shift attention between objects of interest. Toward understanding and being able to predict differences in attentional processing, it is also useful to determine how participants *continually* shifted attention between focal and context objects. For insight on continual use of context information, we have chosen to analyze eye movement patterns between and within objects of interest. To do this, transition percent matrices were created for eye movements moving within and between pre-defined areas of interest on stimulus displays. The transition percentages describe how participants shifted attention between, and within objects. Transition matrices have previously been suggested for the analysis of eye movements between areas of interest (e.g., Stark and Ellis, 1981).

Eye movement patterns can contribute important information about attention to context when facial emotions are interpreted. For example, viewers may share similar statistics with respect to the variables already addressed to date in the literature (i.e., *where participants looked*, *when* they initiated a shift of interest from the focal object, and *how long* they dwelt on focal vs. context objects), but may *differ on how* they scanned in search of information.

### The Present Study

The goal of the present study was to augment previous cross-cultural findings (i.e., Masuda et al., 2008, 2012), by determining how participants from two cultures *continually* shift attention to utilize context, when judging the expression on a focal face. The stimuli, emotion-bearing focal cartoon characters that were flanked on either side by emotion-bearing cartoon characters, were those used by Masuda et al. (2008). The flanking faces in the stimuli provide competing context for the allocation of attention. As we have utilized Masuda’s methodology (Masuda et al., 2008, 2012), it was important first to replicate earlier findings on attention to facial emotion context (see Open Science Collaboration, 2015, for a discussion on the importance of replications in Psychology research). Toward replication, we analyzed the latency of initial eye movements away from the focal face, and proportions of time spent looking at the flanking faces. From Masuda’s work (Masuda et al., 2008, 2012), we expected East Asians to look away from the initially fixated focal face earlier than Westerners, and spend a higher proportion of time looking at flanking faces. Based on the theory that East Asians are more holistic than Westerners in how they attend to visual scenes, we further hypothesized that East Asians would distribute their eye fixations more widely than Westerners over the stimuli. We tested this hypothesis by analyzing the area over which eye fixations were distributed.

Toward determining how East Asians and Westerners *continually* shifted attention to utilize context, we analyzed eye movement patterns between and within interest areas (IA). Eye movement patterns were quantified as proportions of eye movements between, and within focal and context faces. Participants were a multi-ethnic set of American students in USA (i.e., the Western group), and Taiwanese students in Taiwan (i.e., the East Asian group). Given that Masuda et al. (2012) found that Asian and European Canadian students in Canada behave more alike than Japanese students in Japan, all students in the American sample were regarded as Western regardless of ethnicity.

The stimuli were divided into three IA for analyses: Left Flanking Faces (LFF), Focal Face (FF), and Right Flanking Faces (RFF). As such, there were six possible ordered combinations of eye movements between pairs of IA, and three possible combinations of eye movements within IA. The combinations are illustrated in a transition matrix in **Table 1**.

**Table 1:** Eye movement transition matrix.

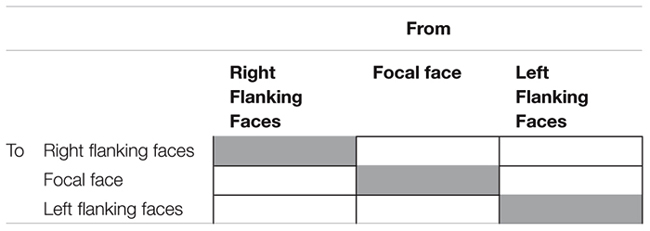

*Transitions between interest areas are shown as open rectangles, and transitions within interest areas are shaded.*

Based on the previously hypothesized importance of context to East Asians (e.g., Nisbett et al., 2001), proportions of eye movements between IA were expected to be higher for East Asians than for Westerners. On the assumption that participants would compare emotions in the flanking areas with the emotion on FF, proportions of eye movements between FF and both flanking areas were expected to be higher than proportions of eye movements between the flanking areas (i.e., LFF to RFF, RFF to LFF). If transitions are similar in pattern between East Asians and Westerners, this would suggest that both cultures utilized similar attention allocation strategies. Conversely, differences in patterns of transition between the cultures would suggest the use of different attention allocation strategies.

## Materials and Methods

### Participants

Nineteen students of various ethnicities (5 African American, 11 Caucasian, 3 other) at the University of Detroit Mercy (UDM), USA participated as Westerners. One of the Westerners had East Asian ancestry. East Asians comprised 22 Taiwanese students at National Dong Hwa University (NDHU), Taiwan. All participants had normal or corrected-to-normal visual acuity, and they were unaware of the purpose of the experiment. The study was conducted in accordance with the Code of Ethics of the World Medical Association (Declaration of Helsinki). Participation occurred with informed consent approved by the relevant Institutional Review Boards at University of Detroit Mercy, and National Dong Hwa University.

### Stimuli

**FIGURE 1:**
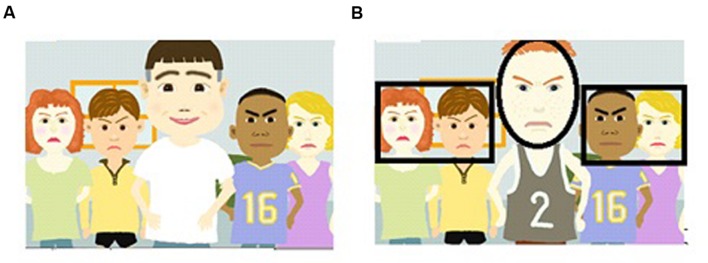
**(A)** An example of stimuli presented to participants. The focal (i.e., central) character was always flanked by four characters. Flanking characters expressed the same emotion, which was different from the emotion on the focal character. **(B)** Face interest areas overlaid (black lines) on a sample display. In the text, the face in the center is referred to as the Focal Face. The two faces to the left were termed Left Flanking Faces, and the two to the right, Right Flanking Faces.

Masuda et al.’s (2008) cartoon stimuli (56 in total) were utilized in the experiment (see **Figure 1A**, for a sample stimulus). Focal faces expressed seven emotions (moderate and intense anger, moderate and intense sadness, moderate and intense happiness, and neutral), and flanking faces, four emotions (anger, sadness, happiness, and neutral). Within a display, all flanking faces expressed the same emotion.

### Apparatus

Stimuli were presented on a computer monitor. Participants in the USA were fitted with a head-mounted Eyelink II (500 Hz sampling) eye tracking headband, controlled by EYETRACK (EYETRACK software^1^). Emotion judgment responses were made with the mouse. For participants in Taiwan, data were collected via Eyelink 1000 (1000 Hz sampling) remote head-free eye tracker, controlled by Experiment Builder software. Emotion judgment responses were made on a keyboard. In Taiwan and in the USA, the average gaze apposition accuracy was about 0.5 deg, and saccades were registered when eye movement exceeded a velocity of about 30 deg/s or an acceleration of about 8000 deg/s^2^.

### Procedure

All participants sat about 51–55 cm from the display such that the stimulus subtended a rectangular area of 30 deg (H) × 21 deg (V). The FF subtended an area of about 69 deg^2^. An experimental session started with a 9-point calibration of the eye tracker. Participants were simply told that the objective of the study was to “Rate the emotions of central person and the intensity of his emotion.” The 56 stimuli were presented in a randomized order for each participant. Each trial started with a fixation point centered on a white screen. With respect to the stimulus displays, the point fixated was located within the chin of focal faces. Experimenter-controlled eye drift correction was performed on the white screen before the start of each trial, to maintain the accuracy of eye tracking. Immediately after drift correction, the fixation point disappeared and the stimulus was displayed for 5 s for participants to judge the emotion on the FF. Pilot testing with Western participants suggested that they were prone to boredom for presentations longer than 5 s. After each stimulus, participants were ask to choose one emotion out of four (i.e., happy, sad, angry, and neutral), and then to choose the level of intensity of that emotion (i.e., a scale from zero to nine). The emotion-identification and level of intensity judgments were utilized to keep participants on task. The results were not relevant for the goals of the present study. At the end of the study, fixation locations on the stimuli were obtained offline by in-house-developed programming codes. Data related to the face areas of stimuli were obtained through Eyelink Dataviewer.

## Results and Discussion

### Verification of On-Task Behavior

**FIGURE 2:**
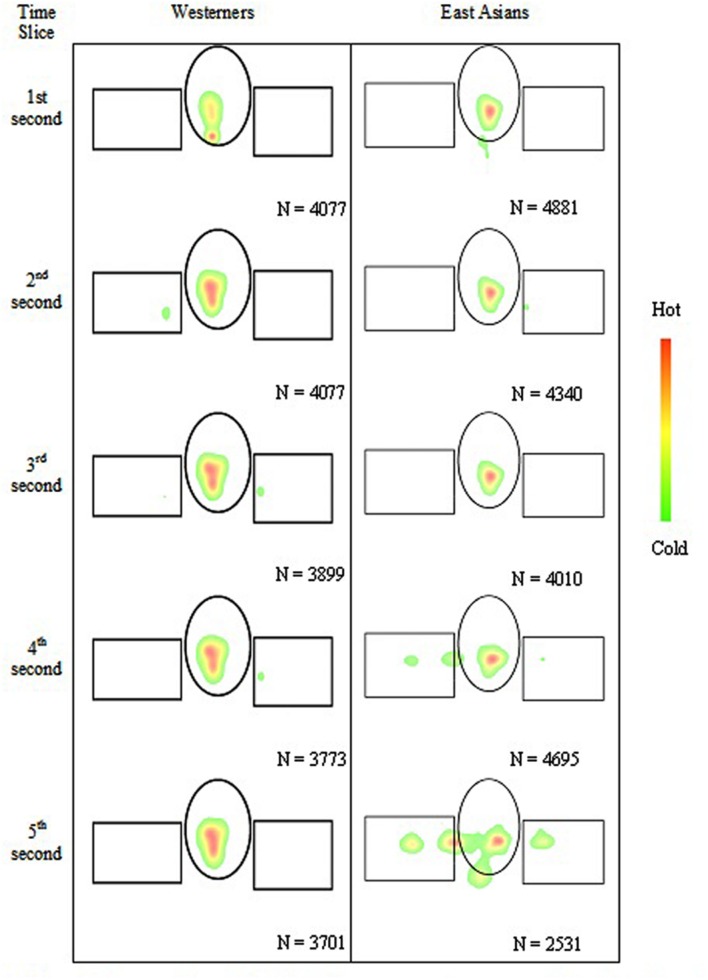
**A green–yellow–red fixation heat map sequence in non-cumulative 1s bins (from top to bottom).** Fixations were smoothed using a 2D Gaussian filter defined by a standard deviation of 0.50 deg. The rectangles and ellipses demarcate the flanking and focal face areas, respectively. The torsos of the stimulus characters are not shown. The figure shows that participants were mostly focused on the face areas (see red hot areas), and not the torsos (not marked) below the faces. Later in the stimulus presentations, East Asians tended to also focus on the flanking faces. *N* indicates the number of fixations utilized in making the heat maps.

Participants’ gaze positions were visible on the experimenter’s monitor. Hence, the experimenter was able to verify that participants were on-task throughout the experiment. Toward verifying on-task behavior in a formal manner, a fixation heat map sequence is presented in **Figure 2**. Fixations were smoothed using a 2D Gaussian filter defined by a standard deviation of 0.50 deg. The figure shows that participants were focused on the face areas, and not the torsos below the faces. **Figure 2** also shows that East Asian participants continued more than Westerners, to look at the flanking faces late into the 5 s stimulus presentation.

Additionally, the high emotion recognition accuracy rate for the Westerners (*M* = 92%, *SD* = 6) suggests that they were on task. Unfortunately recognition accuracy could not confidently be utilized as an additional means of verifying on-task behavior for the East Asian participants, as accuracy for 20 of the 22 East Asians was inadvertently not recorded. However, the accuracy rate for the remaining two East Asians was high (*M* = 87%, *SD* = 8).

### East Asians Distributed Fixations More Widely than Westerners

**FIGURE 3:**
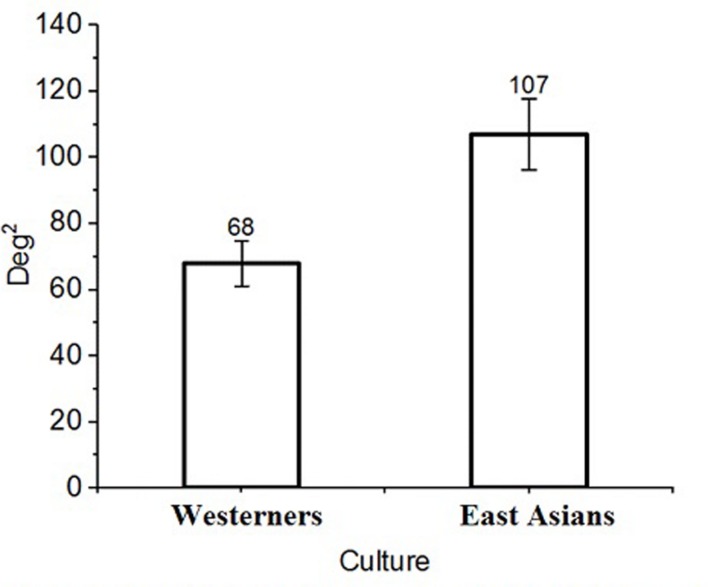
**Bivariate Contour Ellipse Area (BCEA) comparisons.** East Asians had a wider spread of fixation points than Westerners. Error bars represent ±1 SEM.

East Asians, being more holistic in their attention to visual scenes, were expected to distribute their fixations more widely over the stimulus displays than Westerners. Bivariate Contour Ellipse Areas (BCEA) were calculated to quantify the spread of each participant’s eye fixation points over the stimuli. The area (*A*) of each ellipse (see Steinman, 1965) was calculated such that: *A* = 2π1.14 *h*_SD_ *v*_SD_ (1 - *r*^2^)^5^ where *h*_SD_ and *v*_SD_ are standard deviations along the horizontal and vertical meridians, respectively; *r* is the correlation between these two position components. In the present experiment, the ellipse enclosed that portion of the stimuli where 68% eye fixations were located. The mean number of eye fixations per participant that were considered for calculating BCEA were comparable for East Asians (931 fixations) and Westerners (900 fixations), *t*(39) = 0.44, *p* > 0.05, *d* = 0.14. An independent samples *t*-test revealed that, as expected, East Asians A allocated fixations over a wider area (107 deg^2^) than Westerners E (68 deg^2^), *t*(39) = 2.98, *p* < 0.01, *d* = 0.95. The areas are compared in **Figures 2** and **3**. The area viewed by Westerners E (68 deg^2^) was comparable with the size of the FF (69 deg^2^). The findings are consistent with reports of East Asians having a greater number of fixations than Westerners in flanking face areas in similar stimulus displays (Masuda et al., 2008, 2012).

### East Asians Looked Away from the Focal Face Earlier than Westerners

East Asians, being more holistic in their attention to visual scenes, were expected to initiate an eye movement away from the initially fixated FF earlier than Westerners (see Masuda et al., 2008). An independent samples *t*-test was conducted on initial dwell times before gaze was shifted away to a flanking face area. As was expected from Masuda et al. (2008), East Asians (gaze latency = 1550 ms) were quicker by 892 ms, to look away toward the flanking face areas than Westerners (gaze latency = 2442 ms), *t*(39) = 3.26, *p* < 0.01, *d* = 1.04.

### Proportion of Viewing Time on Flanking Faces

**FIGURE 4:**
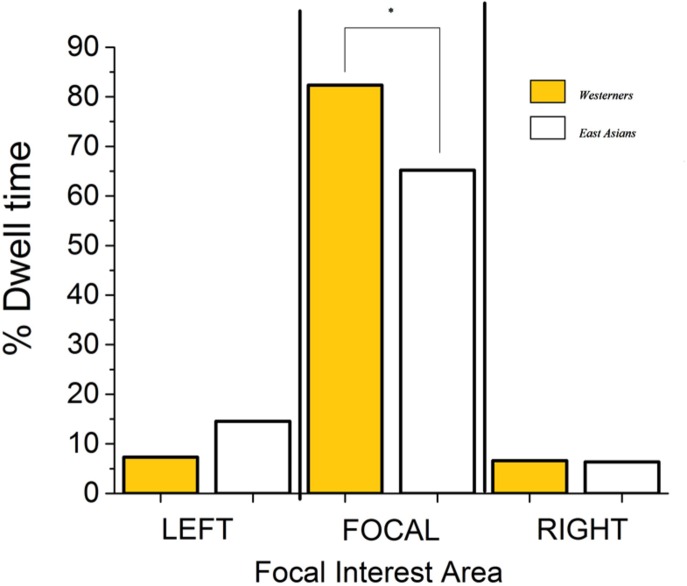
**Percentage of eye dwell time in Areas of Interest.** East Asians spent a significantly lower percent of eye dwell time than Westerners on the focal face. The asterisk denotes *p* < 0.01.

East Asians, being more holistic, were expected to spend more time looking at flanking faces than Westerners (see Masuda et al., 2012). To evaluate how long participants looked at focal and flanking faces, the stimuli were partitioned into three face IA: LFF, FF, and RFF (see **Figure 1B**). It should be noted that only the face areas were of interest in the partitioning of the stimuli. A 2 (Culture of Participants: East Asians vs. Westerners) × 3 (Area of Interest: LFF vs. FF vs. RFF) ANOVA was conducted on percentage of viewing times in these face IA. There was a significant main effect of Culture of Participants, such that Westerners spent a higher percentage of their viewing time (32%) within the face IA of the stimuli than East Asians (29%), *F*(1,39) = 13.55, *p* < 0.01, $\eta_{p}^{2}$ = 0.99. Participants did not spend equal proportions of eye dwell times within the three face IA [11% in LFF, 74% in the FF area, 7% in the Right flanking face area, *F*(2,78) = 432.97, *p* < 0.01, $\eta_{p}^{2}$ = 0.92]. Of primary interest was the significant interaction (shown in **Figure 4**) between Culture of Participants and Area of Interest, *F*(2,78) = 11.91, *p* < 0.01, $\eta_{p}^{2}$ = 0.23. Bonferroni tests (and less conservative Tukey tests also) revealed that Westerners spent a higher percent of viewing time than East Asians within the FF area (*p* < 0.01). This replicates Masuda et al.’s (2012) of Westerners having a higher likelihood of allocating attention to the FF. However, contrary to expectation, Bonferroni tests (and less conservative Tukey tests also) revealed no significant cultural differences in attention to the flanking faces (smallest *p* = 0.17). It may be worthwhile to note that Fisher LSD tests (i.e., with no correction for Type I error) suggested that East Asians spent a significantly higher percent of dwelling time than Westerners on the LFF (*p* = 0.02). The results of this uncorrected analysis match the results of Masuda et al. (2012), who found that East Asians attended more than Westerners to flanking faces. Masuda et al. (2012) did not report corrections for their *a posteriori* multiple *t*-tests. A conservative conclusion is that Westerners and East Asians did not significantly differ in their percentages of eye dwell time on the flanking faces, within our 5 s stimulus presentation window. Less conservatively, it may be reasonable to conclude that there was a trend toward East Asian spending a higher percent than Westerners of eye dwell time on the LFF.

### Cultural Differences in Sequential Eye Movement Behavior

If the strategy of research participants was to compare flanking and focal information to make judgments about the FF, then this was expected to be apparent in eye movement transition percent matrices. Specifically, the proportions of eye movements between the FF and each flanking area was expected to be higher than proportions of flank-to-flank eye movements. With East Asians more likely to utilize context information in cognitive processing (Masuda and Nisbett, 2001; Masuda et al., 2008), eye movement transition proportions were expected to be higher for East Asians than Westerners.

To begin, a first order eye movement transition matrix was constructed for each participant (e.g., percentage of eye movements from the FF area to the LFF). Two kinds of eye movement patterns were of interest: scanning between IA; scanning within an IA. There are six transition states for the question of how participants scanned between IA (e.g., from FF to LFF), and three transition states for the question of how participants scanned within IA (i.e., from FF to FF, from LFF to LFF; from RFF to RFF).

#### Eye Movements between Interest Areas

A 2 (Culture of Participants: East Asians vs. Westerners) × 6 (Eye movement Transition States) ANOVA was conducted on percentage of eye movements made between IA. East Asians were more likely to make eye movements between IA than Westerners (4.7% vs. 2.5% of eye movements made between the IAs), *F*(1,39) = 17.49, *p* < 0.01, $\eta_{p}^{2}$ = 0.31. Eye movement percentages were not the same for all the transition states *F*(5,195) = 85.86, *p* < 0.01, $\eta_{p}^{2}$ = 0.69. For example, eye movements were more likely to be made from the FF to the LFF (6.6% of eye movements) than from the LFF to the RFF (1.0% of eye movements), (Bonferroni correction, *p* < 0.01). Percentages for all transition states are presented in **Table 2**. There was no significant difference (Bonferroni correction, *p* > 0.05) between percentages of eye movements made from the LFF to FF (5.5%) and percentages of eye movements made from the FF to the RFF (4.3%). No difference was found also for eye movement transitions between the left and right flanking faces (LFF to RFF, 0.8%, and RFF to LFF, 1.0%). All other comparisons (with Bonferrroni correction) revealed significant differences (*p* < 0.01).

**Table 2:** Percentage of eye movement transitions between interest areas across cultures.

Transition	Percentage (%)
FF to LFF	6.6
LFF to FF	5.5
FF to RFF	4.3
RFF to FF	3.4
LFF to RFF	0.8
RFF to LFF	1.0


**FIGURE 5:**
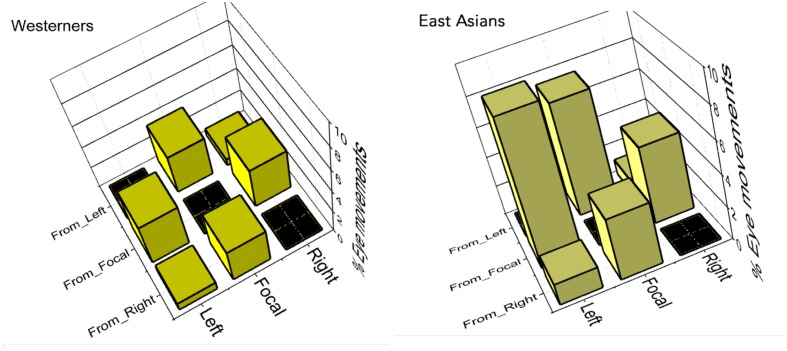
**Eye movement transition percentages between interest areas for Westerners **(Left)** and East Asians **(Right)**.** The matrices show that sequential patterns were not similar between the two cultures.

Most importantly for the purpose of the present work, the Culture of Participants × Eye movement Transition States interaction effect was significant, as shown in **Figure 5**, *F*(5,195) = 18.03, *p* < 0.01, $\eta_{p}^{2}$ = 0.32. Thus, culture influenced sequential eye movement patterns. Simple effect tests of Culture of Participants at levels of Eye movement Transition States (with Bonferroni corrections) showed that East Asians were more likely than Westerners to look sequentially between the LFF and the FF (all *p*s < 0.01), and from the RFF to the LFF (*p* < 0.05).

**FIGURE 6:**
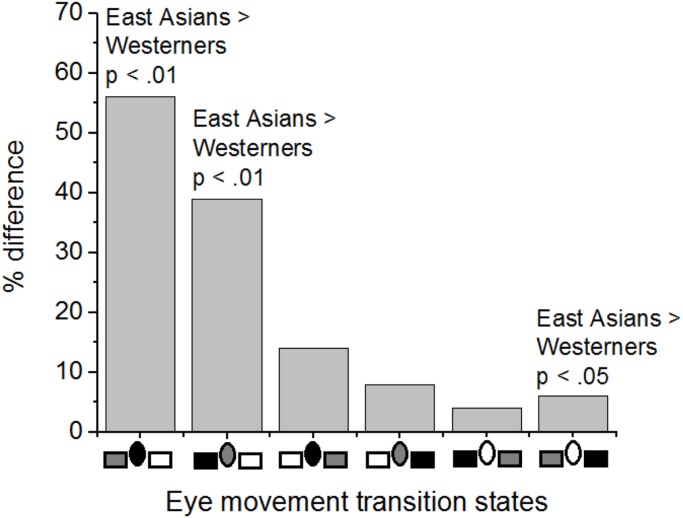
**Cultural differences in eye movement transition percentages between interest areas.** On the *x-*axis, rectangles represent left and right flanking faces, and an ellipse represents the focal face. The three interest areas are configured to depict transitions from the dark-shade area to the light-shade area. For example, in the first column, East Asians made a higher percentage of eye movements than Westerners from the focal face (i.e., dark shade) to the left flanking face area (i.e., light shade).

For simplicity, cultural differences in eye movement transition percentages between IA are illustrated **Figure 6**. Eye movement transitions were most disparate between East Asians and Westerners when gaze was shifted between the FF and LFF.

With respect to a comparison of transition proportions within each culture, simple effect tests of Eye movement Transition States at levels of Culture of Participants were conducted (with Bonferroni corrections). No difference was evident among pairs of transition proportions when Westerners made eye movements between the focal and flanking faces (*p* > 0.05; see **Figure 5**). For East Asians, however, all pairs of transition proportions between FF and the flanks were significantly different (*p* < 0.05; see **Figure 5**), except for pairings of FF and RFF (*p* > 0.05; see **Figure 5**). Together, the results indicate that there were cultural differences in sequential eye movement patterns.

#### Eye Movements within Interest Areas

**FIGURE 7:**
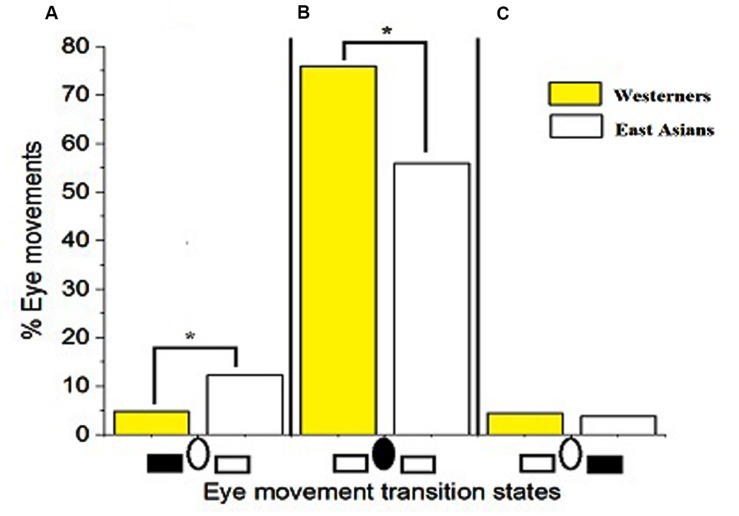
**Cultural differences in eye movement percentages within interest areas.** On the *x-*axis, rectangles represent left and right flanking faces, and an ellipse represents the focal face. The shaded area indicates the area of interest. For example, in Panel A, East Asians made a higher percentage of eye movements than Westerners within the left flanking faces. Asterisks denote *p* < 0.01.

A 2 (Culture of Participants: East Asians vs. Westerners) × 3 (Eye movement Transition States) ANOVA was conducted on percentage of eye movements made within IAs. East Asians were less likely to make eye movements within IAs than Westerners (24% vs. 28% of eye movements made within the IA), *F*(1,39) = 17.84, *p* < 0.01, $\eta_{p}^{2}$ = 0.31. Eye movement percentages were not equal for all the transition states *F*(2,78) = 348.55, *p* < 0.01, $\eta_{p}^{2}$ = 0.90. Eye movements were more likely to be made within the FF (66% of eye movements) than within either of flanking faces (less than 8.6% of eye movements), (Bonferroni correction, *p* < 0.01). The Culture of Participants × Eye movement Transition States interaction effect was significant *F*(2,78) = 14.29, *p* < 0.01, $\eta_{p}^{2}$ = 0.27. Simple effect tests of Culture of Participants at levels of Eye movement Transition States (with Bonferroni corrections) showed that East Asians had lower percentages of eye movements within the FF (*p* < 0.01), but higher percentages of eye movements within the LFF area (*p* < 0.01). No cultural difference was evident for eye movements within the RFF. Cultural differences in eye movement transition percentages within IA are illustrated **Figure 7**.

## General Discussion

Knowledge of how information is attended to in the visual world to make decisions is useful for predicting behavior. In the present study, we have addressed how information was sought to interpret facial expressions. Research by Masuda et al. (2008, 2012) suggests that as East Asians try to interpret the facial expressions, they are more likely than Westerners to incorporate the expressions on flanking faces. Previous eye movement data suggest that East Asians do this by starting to look at the background faces earlier (Masuda et al., 2008) and for longer periods (Masuda et al., 2012) than Westerners. The present study reports evidence in support of these earlier findings. Specifically, similar to Masuda et al. (2008), we found that East Asians initiated eye movements to flanking faces earlier than Westerners. Additionally, both cultures spent an equally low percent of their stimulus-viewing time on RFF, but East Asians exhibited a trend toward spending a higher percentage of time looking at the LFF than Westerners. Although Masuda’s (Masuda et al., 2008, 2012) findings (and the current replications) provide information about *what* East Asians and Westerners looked at, and *when* they executed the first gaze shift from a FF, they say little about *how* participants continually sought information from flanking faces. We addressed this gap by determining cultural differences in the proportions of eye movements made between, and within focal and flanking faces. Not surprisingly, given that the task was to decipher the emotion on the focal face, results indicated that both cultures had the highest proportions of eye movements within the focal face. Assuming East Asians are more holistic in their attention to detail (e.g., Nisbett et al., 2001), proportions of eye movements between IA were expected to be higher for East Asians than for Westerners. On the assumption that participants would compare emotions in the flanking areas with the emotion on FF, proportions of eye movements between FF and both flanking areas (e.g., FF to LFF) were expected to be higher than proportions of eye movements between the two flanking areas (i.e., LFF to RFF, RFF to LFF). As expected, we found that East Asians had a higher proportion of eye movements between IA than Westerners, and proportions of eye movements between FF and both flanking areas were higher than proportions of eye movements between the two flanking areas.

The main finding was that the sequential allocation of attention (with eye movements) to the presented faces was influenced by culture. East Asians scanned less than Westerners within the FF, and more than Westerners within the LFF. East Asians also scanned more than Westerners between the FF and LFF, before reporting the emotion on the FF. This (previously unreported) left-context bias in scanning behavior for East Asians is a topic for further investigation. From the perspective of neuroscience, an appealing explanation for the observed scanning bias may be cerebral hemispheric lateralization. Indeed, neuroimaging findings have shown that the processing of neutral whole faces exhibit right cerebral hemisphere/left visual field dominance (Rhodes, 1985; Yovel et al., 2008; Rossion et al., 2012), and this is especially so for East Asians (Goh et al., 2010). However, no study has monitored cerebral hemispheric responses during the interpretation of emotions in the context of nearby emotions. Such studies would be informative, given that previous studies have found that right lateralization may not be reflected in the processing of all facial emotions (see Najt et al., 2013).

In the present study, the flanking faces all bore the same emotion. From an information-processing perspective, this configuration made one flanking face area redundant for information acquisition. In other words, comparison of one flanking set of faces with the focal face was sufficient. The finding that East Asians scanned mostly between the LFF and the FF supports this argument. Little additional information would have been gained by also scanning between the FF and RFF. The neuroscience and information-processing perspectives are complimentary, in the sense that greater right lateralization in East Asians for faces (Goh et al., 2010) may bias context information acquisition toward the left visual field, if information in the right visual field is redundant.

During emotion processing in the real world, it is not unusual for persons near the target person to express diverse sets of emotions. In future studies, the emotions on flanking faces must be manipulated to determine how information is sought before the focal emotion is reported. Despite the uniformity of emotions on the flanking faces, we have provided evidence that East Asians overtly (with eye movements), and systematically, sought information from surrounding (context) faces at rates higher than Westerners. The findings are important even if eye movement patterns may be specific to visual stimulus sets (e.g., Stark and Ellis, 1981). That is to say, if patterns in a different task than what we have utilized turn out not to match what we have found, this would not hinder our ability to predict cross-cultural attentional performance in the current facial emotion recognition task. Finally, eye movement patterns were different across cultures. The bias toward scanning between FF and LFF demonstrated by East Asians was absent for Westerners (who equally scanned between FF and LFF, and FF and RFF). Differences in eye movement patterns between the cultures suggests the use of different attention allocation strategies, and this may be a step toward supporting the theory that East Asians utilize different cognitive processes than Westerners to interpret visual scenes (e.g., Nisbett et al., 2001; Nisbett, 2003).

### Implications

Our long-term goal is to determine how participants of different cultures continually shift attention between focal and context faces to interpret facial emotions in social settings. The patterns of eye movement in the present study suggest that East Asians more so than Westerners, engaged in *comparisons* of focal and context information in order to make a judgment about the FF. This has implications for simulating scenarios in cross-cultural training, and for improving context awareness in human–computer interactions (e.g., affective computing, AC). For cross-cultural training, trainees may be familiarized with patterns of eye movement toward appreciating the perspective of the other culture. AC involves the development of computational systems that can interpret human affect (Picard, 1997). Given the importance of context to human interactions (and the sensitivity of culture to context), AC systems stand to benefit from implementing context-awareness (Vlachostergiou et al., 2014). Cross-cultural findings on attention to context have the potential to make AC systems culturally competent in their context awareness. Eye movement transition weightings may inform the operation of these systems, as they collect information in real time to judge a user’s emotion.

In the present study, we have utilized first-order eye movement transition matrices (e.g., probability of eye movements from the FF area to the LFF) to determine how participants scanned between a FF and flanking faces, as they judged the emotion on the FF. While these first-order statistics are insightful, predictability of scanning behavior may be improved by utilizing higher-order eye movement transition matrices (e.g., probability of eye movements from the FF area to the LFF, and back to the FF area).

### Future Research Directions

For the interpretation of a focal facial expression in the context of flanking facial expressions, we have replicated evidence that East Asians distribute their eye fixations more widely than Westerners, and start to look away from the FF earlier than Westerners (Masuda et al., 2008, 2012). There was also a trend toward East Asians spending a higher proportion of viewing time than Westerners on flanking faces. Beyond replications, we have provided novel evidence that East Asians are more likely than Westerners to continually shift attention between focal and context faces after the initial shift of attention from the FF. Whereas it was already known that East Asians integrate information from multiple people within a social context when attempting to identify the emotion of a focal person (Masuda et al., 2008, 2012), our findings describe how information may be sought continually, to make decisions. We have suggested that sequential scanning statistics are important because they can provide evidence-based information for simulating scenarios in cross-cultural training, and for implementing culturally sensitive AC systems.

Several limitations of the present study may be addressed by future research. For example, while the accuracy rate for the identification of facial emotions is typically very high across cultures (Ekman et al., 1987), there exists an in-group advantage, such that East Asians may be expected to be more accurate at identifying East Asian than Caucasian facial emotions (see Zhang et al., 2015). The design of the present study could have allowed us to address in-group advantage questions in the context of competing flanking emotions. Unfortunately, given the limited availability of emotion identification data (due to data loss among East Asian participants) context parameters of the in-group advantage could not presently be addressed.

Another issue of interest is decision-making time. Movement of the eyes allow the viewer to process sequentially, the details of spatially distributed stimuli (e.g., faces) in the visual field. In the present study, we had access to when, where, and how the eyes were moved over the face stimuli (e.g., **Figures 2** and **5**). However, given that stimulus presentation duration was fixed at 5 s, the design of the study did not allow for access to *when* the emotion identification decision was made. It is possible that there were differences in the average time required to identify the emotion. Hence, some of the eye movement behavior observed in the present study may reflect differential free viewing after the emotion had been identified. In the future, cultural differences in emotion identification times may be addressed by instructing participants to terminate the stimulus presentation as soon as the emotion is identified (e.g., Ito et al., 2012).

A third issue of concern is the locus of initializing looking behavior (e.g., Greene, 2008). In the present study, participants always initiated their looking behavior from the focal face. In the real world, the face of interest may not be the initial locus of attention. As a complement to the issues addressed in the present study, it would be useful in the future to determine eye movement behavior when attention is initially on a flanking face. While East Asians do appear to attend to context more than Westerners (as we have shown; see also Masuda et al., 2008, 2012), they (East Asians) like Westerners, do also prioritize focal stimuli (as we have shown; see also Masuda et al., 2012). It is not known how culture will affect sequential eye movement behavior when the initial fixation is not on the face of interest.

Finally, cross-cultural research has provided ample evidence to suggest that human groups do not all process information the same way. While the direction of gaze does not necessarily indicate a viewer’s locus of covert attention, it does, however, indicate how the eyes are positioned (i.e., foveated) to maximize the acquisition of spatially distributed information. An important issue yet to be well addressed in cross-cultural studies is the utilization of extrafoveal information. If human groups do indeed differ in the way they fundamentally acquire information in the visual world, then it is important to determine differences in foveal and extrafoveal mechanisms involved, toward anticipating (and perhaps managing) responses that culturally different viewers make to identical visual stimuli.

## Author Contributions

All authors listed, have made substantial, direct and intellectual contribution to the work, and approved it for publication.

## Conflict of Interest Statement

The authors declare that the research was conducted in the absence of any commercial or financial relationships that could be construed as a potential conflict of interest.

## Footnotes

1 http://www.umass.edu/psychology/div2/eyelab/
